# An improved time reversal mirror based on standard linear frequency modulation waveform

**DOI:** 10.1038/s41598-020-79884-w

**Published:** 2021-01-08

**Authors:** Yongkang Wang, Han Zhang, Huiling Li, Jianfeng Zheng, Liang Guo

**Affiliations:** 1 State Key Laboratory of Nonlinear Mechanics, Institute of Mechanics, Chinese Academy of Sciences, Beijing, 100190 China; 2 Key Laboratory of Noise and Vibration, Institute of Acoustics, Chinese Academy of Sciences, Beijing, 100190 China; 3 State Key Laboratory of Acoustics, Institute of Acoustics, Chinese Academy of Sciences, Beijing, 100190 China; 4 University of Chinese Academy of Sciences, Beijing, 100049 China; 5 School of Engineering Science, University of Chinese Academy of Sciences, Beijing, 100049 China; 6 School of Mechanical Engineering, Changzhou University, Changzhou, 213164 China

**Keywords:** Acoustics, Engineering

## Abstract

Time reversal mirror (TRM) technology is the adaptive focusing method evolved from the phase conjugate method in optics. Conventional incentive method in TRM technology is a narrow pulse signal with a high bandwidth. In this paper, the autocorrelation property of the TRM was proved from the time-reversal symmetry of the wave equation. The linear frequency modulation (LFM) signal is adopted as the exciting signal in the TRM, which gives the dual autocorrelation function waveform, including the exciting signal and transport channel response. Theoretical results show that the peak value of the transducer array’s focusing signal is determined by the pulse width of the LFM signal and the number of array elements. In addition, the adaptive filtering deconvolution method is used to precisely regulate the input signal to ensure that the final detecting signal is the expected LFM waveform, which eliminates the effect of the transport channel and enhances matched filtering effects. The results hold great theoretical significances for the development of TRM technology in ultrasonic detection.

## Introduction

**Figure 1:**
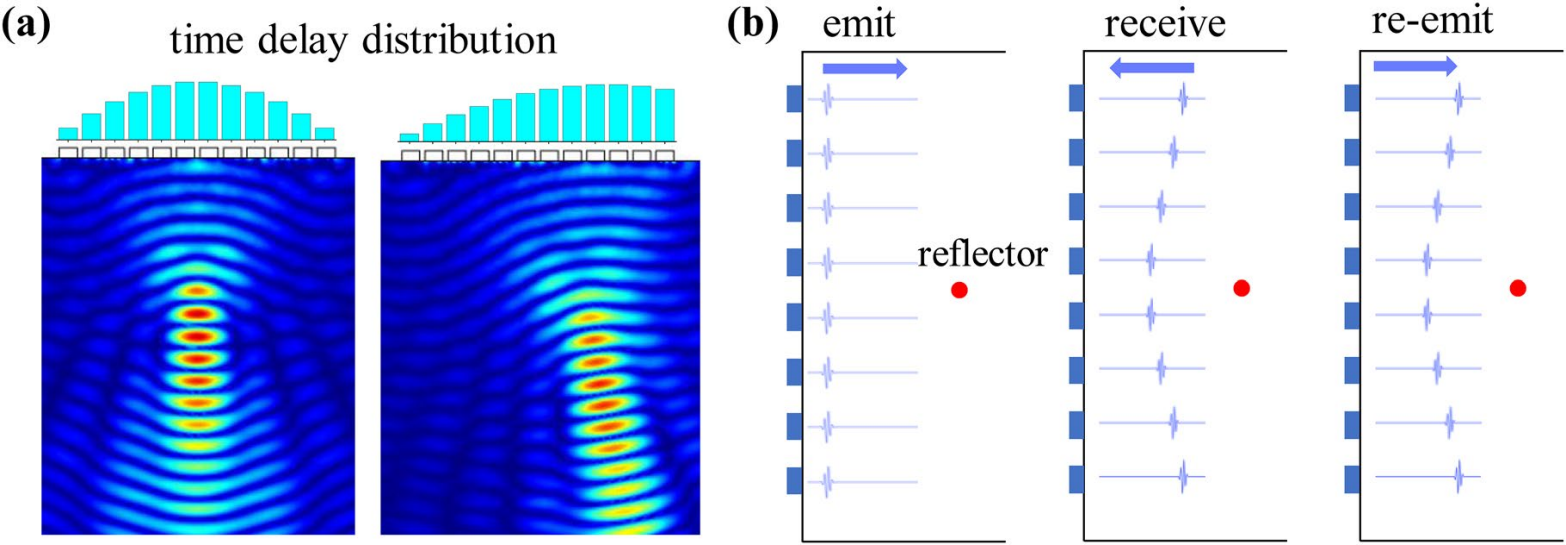
The detecting technology of the phased array. (**a**) The focusing and deflection of the acoustic beam under electronic control. (**b**) Adaptive focusing processing of the TRM.

Ultrasonic non-destructive testing (NDT) technology is able to detect interior of structures effectively without destroying the measured objects, which promotes its broad applications in aerospace, city infrastructure, mechanical engineering, and other fields that need defect detection and quality monitoring^1–6^. In general, the ultrasonic phased array realizes the acoustic beam focusing and deflection by the independent phase delay excitation of each element to achieve excellent detecting performance (Fig. 1a)^3–7^. However, the ultrasonic phased array based on delay excitation is difficult to realize the acoustic beam controlling without prior knowledge for inhomogeneous material^8^.

Time reversal mirror (TRM) technology has characteristics of the compensating multi-path effect and adaptive focusing, which has attracted extensive research interests for the past few years. TRM technology, which is regarded as a generalized phase conjugate mirror method, is derived from the phase conjugate method in optics^9–11^. The TRM consists of transducers array, allowing the incident acoustic field to be sampled, time-reversed, and re-emitted (Fig. 1b). This process realizes the matched filtering to the inhomogeneous propagation transfer function between the array and the focal target, and all time reversal signals are simultaneously focused on the acoustic source^8–12^. The matched filtering is equivalent to the autocorrelation processing of the transport channel function, which has the optimal signal-to-noise (SNR) output^8,13^. While multiple reflectors exist in the medium, the iterative algorithm of the time reversal process can realize the adaptive focusing at the most reflective target^9,14^. Whatever the measured object is solid, fluid or stratified medium, the TRM always shows excellent focusing performance^15–19^. In recent years, TRM technology has showed great research significances and application values in the fields of defect detection of composite materials, medical diagnosis and treatment, underwater target recognition and so on^18–25^. However, The conventional exciting signal widely used in the TRM is a narrow pulse signal with a high bandwidth, which can obtain the high axial resolution and detect micro-size defects^8,14,26^. While the SNR and detecting range are required to be higher, the pulse width needs to be increase, which inevitably affect the axial resolution of the detecting signal.

The time bandwidth product of the conventional pulse signal is a constant, which leads to the fact that the pulse width and the bandwidth are mutually restricted. The larger pulse width represents the higher input energy while the larger bandwidth represents the higher axial resolution, which involves an inevitable contradiction. Inspired by radar signal processing technology, encoded signals based on pulse compression technology has broken the limitation of the time bandwidth product^27,28^. As the controllable and special signal with a large time bandwidth product, the linear frequency modulation (LFM) signal is initially used in the field of radar system, and then gradually spreads to ultrasonic applications^26,28–31^. The application of the LFM excitation improves the SNR and detecting depth of ultrasonic detection and overcomes some defects of conventional excitations^26^. In a ultrasonic detecting system, due to the limitation of the transducer’s bandwidth, the detecting echo signal obtained by LFM exciting signal is always distorted, which results in serious reduction of the matched filtering effect. What’s more, distortions of the detecting signal becomes more serious with the increasing of the bandwidth of the exciting signal^26,32^. Testing and recording the transmitted and received signals of the determined detecting system under the impulse excitation, and then the exciting signal corresponding to the desired detecting signal can be obtained by adaptive filtering deconvolution method, which can eliminate the influence of transport channel response^32^.

Based on the time-reversal symmetry of the acoustic wave equation, the autocorrelation property of matched filtering for TRM technology is demonstrated and then the theoretical model of TRM technology based on the LFM signal is established in this study. The results show that the final detecting signal is in form of the dual autocorrelation function, including the autocorrelation functions of the exciting signal and the transport channel response. The transport channel response cannot be regulated, but the parameters of the LFM exciting signal can be adjusted and designed in advance. Compared with the sharp pulse exciting signal, the focusing signal’s peak value of the phased array depends on the pulse width of the LFM exciting signal and the number of the array elements, which is controllable and adjustable. The adaptive filtering deconvolution method is adopted to eliminate the influence of the transport channel response, which ensures that the detecting signal of the time-reversal signal is the standard LFM waveform and improves the matched filtering effects. Combined with the adaptive filtering deconvolution method, the improved TRM technology effectively improves the SNR and the performance of ultrasonic detection.

## Results and discussion

### Time-reversal symmetry and autocorrelation

Generalized wave equations are a set of differential equations derived from Maxwell’s equations to describe various wave phenomena in nature. The acoustic wave equation reflects the basic relation satisfied by the acoustic variables. Supposing that (1) the acoustic propagation medium is the continuous fluid medium, (2) the acoustic propagation medium is inhomogeneous and lossless, and (3) the acoustical waves are small amplitude waves, then the linear homogeneous wave equation is determined by the simultaneous equations of continuity, motion and state

1 $$\nabla^{2} p\left( {r,t} \right) - \frac{1}{{c^{2} \left( r \right)}}\frac{{\partial^{2} p\left( {r,t} \right)}}{{\partial t^{2} }} = 0,$$

where $\nabla^{2}$, $p\left( {r,t} \right)$, $c\left( r \right)$, $r$, $t$ represent the Laplace operator, the acoustic pressure field, the acoustic velocity in the medium, spatial coordinates, and time coordinates, respectively. Since only the second order time derivative exists in the wave equation, if $p\left( {r,t} \right)$ is a solution to the wave equation, then $p\left( {r, - t} \right)$ is also a solution, which are symmetric solutions of time inversion. This property is the time-reversal symmetry of the wave equation that indicates: if the acoustic pressure field $p\left( {r,t} \right)$ is obtained by the reflection, refraction or scattering passing through the propagating medium from the point acoustic source, then there must be the acoustic pressure field $p\left( {r, - t} \right)$, which reaches the initial point acoustic source along the same path. In general, time reversal in the time domain $p\left( {r,t} \right) \to p\left( {r, - t} \right)$ corresponds to phase conjugation in the frequency domain $p\left( {r,\omega } \right) \to p^{*} \left( {r,\omega } \right)$ for the real signal $p\left( {r,t} \right)$ (details are provided in section S1 of the Supporting Information).

We analyze the autocorrelation of the TRM in time domain. If the pulse exciting signal of an element in the phased array is $\delta \left( t \right)$, then the received signal at the reflector in the propagation medium can be expressed as

2 $$y\left( t \right) = \delta \left( t \right) \otimes h\left( t \right),$$

where $h\left( t \right)$ is the transport channel response between the array and the reflector and $\otimes$ represents the convolution symbol. The received signal $y\left( t \right)$ is reversed in time and transmitted from the array element again as

3 $$y_{TR} \left( t \right) = y\left( { - t} \right) = \delta \left( { - t} \right) \otimes h\left( { - t} \right).$$

The second received signal at the reflector again is

4 $$y_{TR} ^{\prime}\left( t \right) = \delta \left( { - t} \right) \otimes h\left( { - t} \right) \otimes h\left( t \right).$$

The process of time reversal has the characteristic of spatial matched filter, which is equivalent to autocorrelation processing only for the transport channel response. This property benefits from the time inversion symmetry of the wave Eq. (1). Autocorrelation processing can eliminate the multipath effect of the propagating path, extract the useful signal and improve the SNR (details are provided in section S2 of the Supporting Information). No matter what the channel impulse response is, the result of the convolution in Eq. (4) is maximized at $t = 0$. The maximum value is written as

5 $$A_{P} = \int {h^{2} \left( t \right)} dt,$$

which is same as the energy of the channel response signal $h\left( t \right)$.

In this study, we use the LFM signal $s\left( t \right)$ instead of the sharp pulse signal as the exciting signal, and the received signal at the reflector can be expressed as

6 $$y_{LFM} \left( t \right) = s\left( t \right) \otimes h\left( t \right).$$

The received signal $y_{LFM} \left( t \right)$ is reversed in time and transmitted from the array element again as

7 $$y_{TR\_LFM} \left( t \right) = y_{LFM} \left( { - t} \right) = s\left( { - t} \right) \otimes h\left( { - t} \right),$$

and then the second received signal at reflector again is

8 $$y^{\prime}_{TR\_LFM} \left( t \right) = s\left( { - t} \right) \otimes h\left( { - t} \right) \otimes h\left( t \right).$$

The received signal at the reflector after passing through the matched filter becomes

9 $$y{^{\prime\prime}}_{TR\_LFM} \left( t \right) = \left[ {s\left( { - t} \right) \otimes s\left( t \right)} \right] \otimes \left[ {h\left( { - t} \right) \otimes h\left( t \right)} \right]{ = }R_{ss} \left( t \right) \otimes R_{hh} \left( t \right),$$

which is equivalent to convolving the autocorrelation function $R_{hh} \left( t \right)$ of the transport channel response with the autocorrelation function $R_{ss} \left( t \right)$ of the LFM exciting signal. The $s\left( t \right)$ and $s\left( { - t} \right)$ are a pair of time reversal signals, while the signal $h\left( t \right)$ and $h\left( { - t} \right)$ are also a pair of time reversal signals. Thus we define the final detecting signal $y{^{\prime\prime}}_{TR\_LFM} \left( t \right)$ as the dual autocorrelation function in this study. The transport channel response is invariant in a certain detecting system. The parameters of the LFM exciting signal can be designed in advance, which ensures that the peak value of the final pulse compression signal is controllable. The result of convolution in Eq. (9) maximizes at $t = 0$ and the maximum value is written as

10 $$A_{LFM} = \int {h^{2} \left( t \right)} \cdot s^{2} \left( t \right)dt,$$

which depends on the transport channel response $h\left( t \right)$ and the input LFM signal $s\left( t \right)$.

### The LFM signal based on pulse compression

As the widely used method in radar signal processing system, pulse compression technology realizes both high input energy of long pulse and high axial resolution of narrow pulse, which can resolve the contradiction between pulse width and bandwidth. In particular, as the signal with a large time bandwidth product commonly used in pulse compression technology, pulse width and bandwidth of the LFM signal are convenient for designing and adjusting. The time domain expression of the LFM signal is

11 $$s\left( {\text{t}} \right) = rect\left( \frac{t}{T} \right)e^{{j2{\uppi }\left( {f_{0} t + \frac{K}{2}t^{2} } \right)}} \left( {\left| t \right| \le \frac{T}{2}} \right),$$

where ${\text{f}}_{0}$, $B$, $T$, $K = \frac{B}{T}$ represent the central frequency, the bandwidth, the pulse width, and the slope of frequency modulation, respectively. The central frequency ${\text{f}}_{0}$ is equal to the central frequency $f_{tr}$ of the transducer. $rect\left( \frac{t}{T} \right)$ is the rectangular windows signal, which is expressed as

12 $$rect\left( \frac{t}{T} \right) = \left\{ {\begin{array}{*{20}c} {1, \left( {\left| t \right| \le \frac{T}{2}} \right)} \\ {0,\left( {other{{s}}} \right)} \\ \end{array} } \right..$$

The instantaneous frequency of the LFM signal is obtained as

13 $$f\left( t \right) = f_{0} + Kt,$$

which indicates that the instantaneous frequency $f\left( t \right)$ is a linear function of $t$, and the bandwidth is $B = KT$.

The LFM signal is processed of autocorrelation by matched filter at the receiver, which can extract useful signal and maximize SNR (details are provided in section S3 of the Supporting Information). The time domain expression of the matched filter is

14 $$g\left( t \right) = s^{ * } \left( { - t} \right).$$

While the LFM signal $s\left( t \right)$ only contains the real part, the pulse response of the matched filter $g\left( t \right)$ is the time reversal waveform of the LFM signal. The corresponding acoustic pressure fields of the above time reversal signals all satisfy the same wave Eq. (1) due to the time-reversal symmetry, thus the convolution result of them is a function of the signal $s\left( t \right)$ and itself (i.e. autocorrelation function). Then the waveform of the LFM signal $s\left( t \right)$ after passing through the matched filter is

15 $$s_{0} \left( t \right) = s\left( t \right) \otimes g\left( t \right){ = }\sqrt {B \cdot T} \cdot \frac{{\sin \left( {{\pi B}t} \right)}}{{{\uppi }Bt}} \cdot rect\left( \frac{t}{2T} \right) \cdot e^{{j2{\uppi }f_{0} t}} .$$

The envelope waveform of the compressed signal $s_{0} \left( t \right)$ is written as

16 $$S_{0} \left( t \right) = \sqrt {B \cdot T} \cdot Sa\left( {{\uppi }Bt} \right) \cdot rect\left( \frac{t}{2T} \right).$$

**Figure 2:**
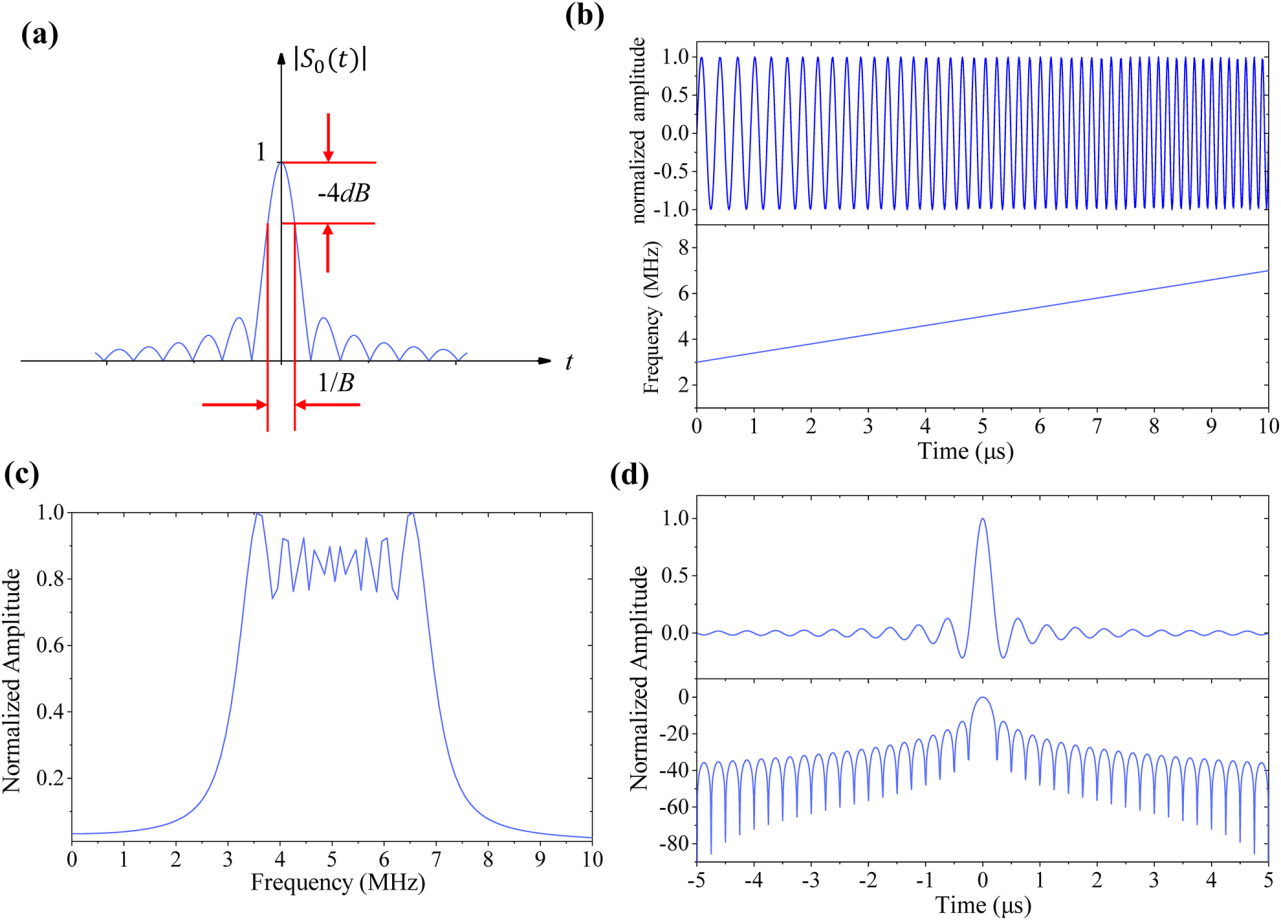
The LFM signal with the time bandwidth of 40. (**a**) The definition of pulse compression signal’s pulse width. (**b**) Time domain waveform and instantaneous frequency of the LFM signal. (**c**) The amplitude-frequency characteristic curve of the LFM signal. (**d**) The time domain waveform of the LFM signal after pulse compression (the top part) and its corresponding signal after logarithm (the bottom part).

While solution $t_{1}$ satisfies ${\uppi }Bt_{1} = \pm {\uppi }/2$ (the − 4 dB main lobe width of the envelope signal, as shown in Fig. 2a), the pulse width of the compressed signal is $\tau = 2t_{1} = \frac{1}{B}$, which is approximate to the axial resolution of the pulse compression signal. And the compression ratio is $D = \frac{T}{{\uptau }} = BT$, which is equivalent to the time bandwidth product.

The LFM signal breaks the limitation of the time bandwidth product of conventional pulse exciting signals (details are provided in section S4 of the Supporting Information). The pulse width $T$ and the bandwidth *B* of the LFM signal, which correspond to the input energy and the axial resolution, are very convenient for designing. For example, when the pulse width $T$, the central frequency $f_{0}$, and the bandwidth $B$ are chosed as 10 μs, 5 MHz, and 4 MHz respectively, the time domain waveform, the instantaneous frequency (Fig. 2b), the amplitude-frequency characteristic curve of the LFM signal (Fig. 2c), and the time domain waveform of the pulse compression signal (Fig. 2d) are obtained from the above theoretical equations. In the bottom part of Fig. 2d, it is assumed that the time coordinate corresponding to first lobe value is $t_{flv}$ and the $t_{flv}$ satisfies the equation ${\uppi }Bt_{flv} = \tan ({\uppi }Bt_{flv} )$ because of the $Sa$ function in Eq. (16). Then the first lobe value is obtained as

17 $$A_{flv} = 20 \cdot \log_{10} \left( {\frac{{S_{0} \left( {t_{flv} } \right)}}{{S_{0} \left( 0 \right)}}} \right) = - 13.26\,{\text{dB}}{.}$$

The sidelobe level can be further reduced by weighting the window function^33–35^. The time bandwidth product $D$ is raised to 40 here and the signal with a larger time bandwidth product is also available if need be.

### Design of LFM signal parameters

**Figure 3:**
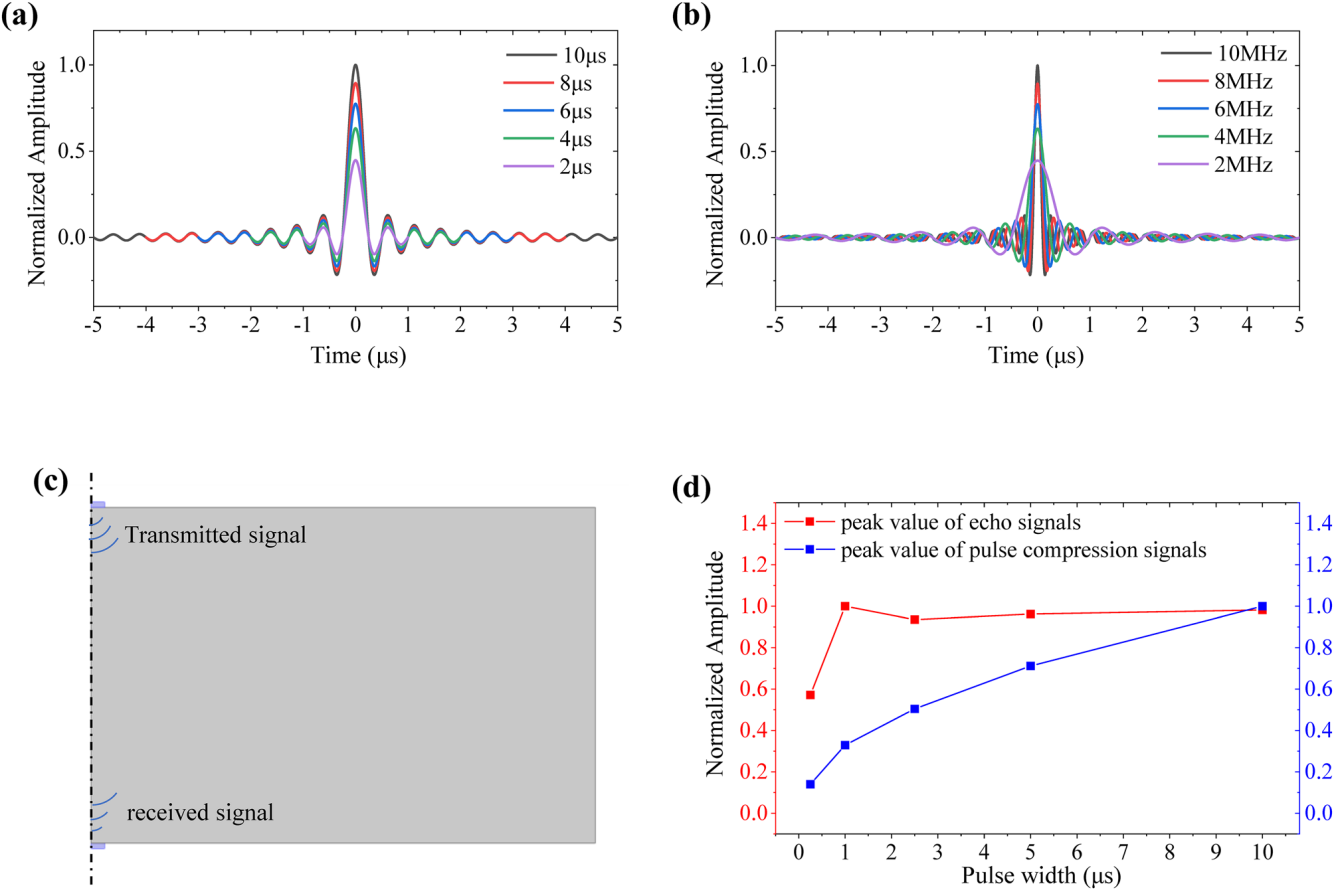
Parameter analysis of the LFM signal. (**a**) Pulse compression signals with different pulse widths of LFM signals (*B* = 4 MHz). (**b**) Pulse compression signals with different bandwidth of LFM signals (*T* = 10 μs). (**c**) The finite element model for transmission and reception of the LFM signals. (**d**) Normalized peak values of the echo signals and pulse compression signals with different pulse widths in FEM.

As one of the LFM signal’ parameters, the central frequency is generally equal to the central frequency of the transducer. Thus the most two important parameters are pulse width $T$ and spectrum bandwidth $B$. The central frequency of the LFM signal is chosen as 5 MHz here. Figure 3a shows that the peak value of the compressed signal positively correlated with the initial pulse width $T$ while bandwidth $B$ is fixed (4 MHz). While pulse width is increased from 0.25 to 10 μs (i.e. the time bandwidth product is increased from 1 to 40), the peak value of the pulse compression signal is improved by 16.2 dB $\left(20 \cdot \log_{10} \left( {\sqrt {\frac{0.25 \times 4}{{10 \times 4}}} } \right)\right)$ according to the Eq. (16). Figure 3b shows that the main lobe width decreases with the improvement of the bandwidth $B$. The smaller main lobe width represents the higher axial resolution. While the bandwidth is increased from 1 to 10 MHz (i.e. the time bandwidth product is increased from 10 to 100 with the fixed pulse width $T$ = 10 μs), the axial resolution is raised 10 times approximately. In general, the bandwidth of the LFM signal, which is matched with the bandwidth of the transducer, cannot be infinite in ultrasonic detection.

The finite element method (FEM) is adopted in the commercial software package COMSOL Multiphysics 5.4 and is used to simulate the transmission and reception of the LFM signals with different pulse widths. The FEM model is shown in Fig. 3c and the spatial dimension of the two dimensional axisymmetric is adopted here. The transmitted transducer is at the top, the received transducer is at the bottom, and the transport medium is in the middle. The transmitted transducer is excited by LFM signals, and the received transducer at the bottom is used for receiving signals by a suspension potential setting. Before the matched filtering, the peak values of received signals of LFM signals with different pulse widths are almost the equal, as shown in Fig. 3d. The low value of the first point may be because the pulse width is too narrow to completely exciting the transducer’s vitration in FEM. After passing through the matched filter, the pulse compression signal with a larger pulse width has a larger peak value, as shown in Fig. 3d. It is verified that the parameters design of the LFM signal can effectively improve the peak value of the pulse compression signal and then improve the SNR according to the simulation results.

### Focusing characteristic of TRM based on the LFM signal

**Figure 4:**
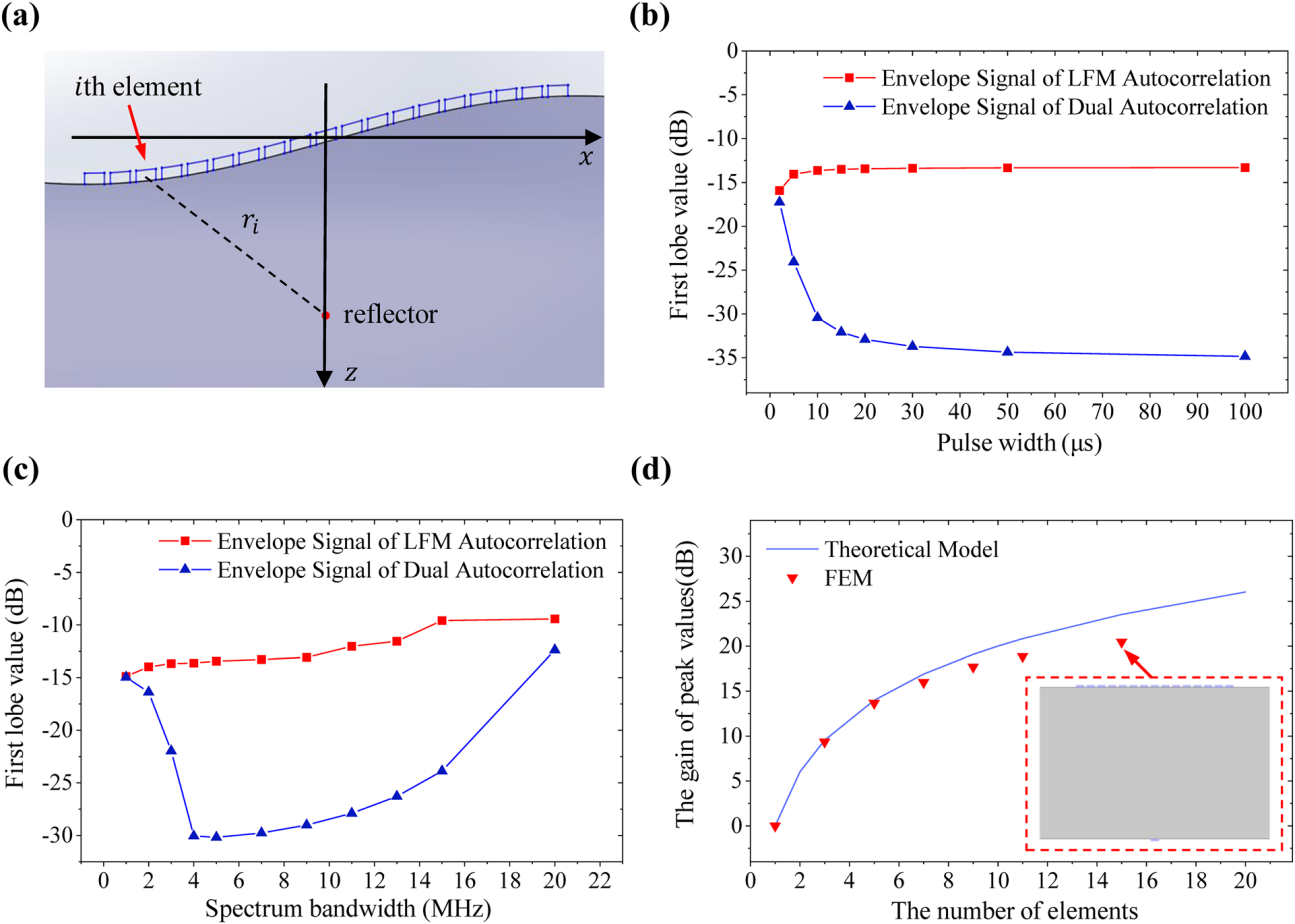
The TRM based on the LFM signal. (**a**) Schematic diagram of the linear phased array on a curve surface. (**b**) The first lobe values of the envelope signal of the LFM autocorrelation function and the dual autocorrelation function with different pulse widths. (**c**) The first lobe values of the envelope signal of the LFM autocorrelation function and the dual autocorrelation function with different spectrum bandwidths. (**d**) The peak value gain of the phased array with the different number of elements.

The TRM can realize the adaptive focusing of the acoustic beam in the measured medium. Conventional incentive signal to the TRM is the excitation of a narrow pulse signal to obtain the excellent axial resolution. In this study we adopt the above LFM signal with a large time bandwidth product instead of the narrow pulse signal to improve the input energy of the transmitted signal. A linear flexible phased array with *N* elements is attached to a curved measured object. Each array element is regarded as a point acoustic source, and the distance between the $i$ th element and the reflector is $r_{i}$ (Fig. 4a). The first transmitted signal of each array element is $s\left( t \right)$ and the received signal at the reflector is

18 $$y_{i} ^{\prime}\left( t \right) = \left\{ {\begin{array}{*{20}c} {s\left( {t - \frac{{r_{i} }}{v}} \right) \otimes h_{i} \left( {t - \frac{{r_{i} }}{v}} \right)\left( {\left| {t - \frac{{r_{i} }}{v}} \right| \le \frac{T}{2}} \right)} \\ {0\left( {\left| {t - \frac{{r_{i} }}{v}} \right| > \frac{T}{2}} \right)} \\ \end{array} } \right.,$$

where $v$ and $h_{i} \left( t \right)$ represent the acoustic velocity in the medium and transport channel response between the $i$ th element and the reflector. After time reversal $T_{r}$ of the received signal, the signal re-emitted from the $i$ th array element is expressed as

19 $$y_{i}^{TR} \left( t \right) = \left\{ {\begin{array}{*{20}c} {s\left( {t - \left( {T_{r} - \frac{{r_{i} }}{v}} \right)} \right) \otimes h_{i} \left( {t - \left( {T_{r} - \frac{{r_{i} }}{v}} \right)} \right)\left( {\left| {t - \left( {T_{r} - \frac{{r_{i} }}{v}} \right)} \right| \le \frac{T}{2}} \right)} \\ {0\left( {\left| {t - \left( {T_{r} - \frac{{r_{i} }}{v}} \right)} \right| > \frac{T}{2}} \right)} \\ \end{array} } \right..$$

The signal at the reflector received again is

20 $$y_{i}^{TR^{\prime}} \left( t \right) = \left\{ {\begin{array}{*{20}c} {s\left( {t - T_{r} } \right) \otimes h_{i} \left( {t - T_{r} } \right) \otimes h_{i} (t)\left( {\left| {t - T_{r} } \right| \le \frac{T}{2}} \right)} \\ {0\left( {\left| {t - T_{r} } \right| > \frac{T}{2}} \right)} \\ \end{array} } \right..$$

From the Eq. (20), it can be concluded that the signal arriving at the reflector is independent of the distance $r_{i}$, i.e. the signal secondly emitted by each array reaches the reflector at the same time. The received signal at the reflector contains the convolution of the transport channel response $h_{i} (t)$ and its time reversal signal $h_{i} \left( {t - T_{r} } \right)$, which are two solutions satisfying the time-reversal symmetry of the same wave equation and ensure the autocorrelation property. The received signal $y_{i}^{TR ^{\prime}}\left( t \right)$ at the reflector from each array is superimposed and then the superimposed signal after the matched filter is obtained as

21 $$y_{sum}^{TR} \left( t \right) = s\left( t \right) \otimes \sum\limits_{i = 1}^{N} {y_{i}^{TR^{\prime}} \left( t \right)} = \left\{ {\begin{array}{*{20}c} {s\left( {t - T_{r} } \right) \otimes s\left( t \right) \otimes \sum\limits_{i = 1}^{N} {h_{i} \left( {t - T_{r} } \right) \otimes h_{i} (t)} \left( {\left| {t - T_{r} } \right| \le T} \right)} \\ {0\left( {\left| {t - T_{r} } \right| > T} \right)} \\ \end{array} } \right..$$

It can be found that the superimposed signals at the reflector contains the autocorrelation functions of the LFM exciting signal $s\left( t \right)$ and the transport channel response $h_{i} \left( t \right)$ of each array. Substitution of Eq. (11) into Eq. (21) gives

22 $$y_{sum}^{TR} \left( t \right) = \left\{ {\begin{array}{*{20}c} {\left[ {\sqrt {B \cdot T} \cdot rect\left( {\frac{{t - T_{r} }}{2T}} \right) \cdot Sa\left( {{\uppi }B\left( {t - T_{r} } \right)} \right)} \right] \otimes \sum\limits_{i = 1}^{N} {h_{i} \left( {t - T_{r} } \right) \otimes h_{i} (t)} \left( {\left| {t - T_{r} } \right| \le T} \right)} \\ {0\left( {\left| {t - T_{r} } \right| > T} \right)} \\ \end{array} } \right..$$

There is no distance parameter $r_{i}$ in the superposition signal $y_{sum}^{TR} \left( t \right)$, and all signals reach the focal point at the same time. The superposition signal includes two parts. One part is the autocorrelation function waveform of the LFM signal. The larger the pulse width of the LFM signal represents the higher the peak value of superposition signal. The other part is the superposition of the autocorrelation function of the transport channel response of each array. As the number of array elements increases, the peak value of the superposition signal is also greatly improve.

The impulse response of the transport channel is set as the Gaussian function

23 $$H\left( f \right) = e^{{ - {\upalpha }\left( {\frac{{f - f_{tr} }}{{B_{tr} }}} \right)^{2} }} ,$$

where $f_{tr} = 5{\text{MHz}}$ and $B_{tr} = 3{\text{MHz}}$ represent the central frequency and bandwidth of the Gaussian function and $\alpha$ is a constant of 4ln2. Considering a single element merely, from Eq. (21) the final detecting signal is obtained as

24 $$y_{sum}^{TR} \left( t \right) = s\left( {t - T_{r} } \right) \otimes s\left( t \right) \otimes h\left( {t - T_{r} } \right) \otimes h(t).$$

The central frequency of the LFM signal $s\left( t \right)$ is chosen as 5 MHz here. $y_{sum}^{TR} \left( t \right)$ in Eq. (24) is regarded as the dual autocorrelation envelope signal and $s\left( {t - T_{r} } \right) \otimes s\left( t \right)$ is regarded as LFM autocorrelation envelope signal.While the bandwidth of the LFM signal is fixed at 4 MHz and pulse width varies from 2 to 100 μs, the first lobe values of the LFM autocorrelation envelope signal and the dual autocorrelation envelope signal in Eq. (24) are compared in Fig. 4b. With the increase of the pulse width, the first lobe value of the envelope waveform of the LFM autocorrelation function remains about − 13.3 dB, which is almost unchanged and close to the theoretical value − 13.26 dB in Eq. (17). And the first lobe value of the envelope waveform of the dual autocorrelation function decreases sharply and tends to be stable while the pulse width increases. As the pulse width reaches 100 μs, the first lobe value of the envelope waveform of the dual autocorrelation function decreases to − 34.85 dB. The reason is that the envelope waveform of the Gaussian signal is the function with a zero sidelobe. Compared with the envelope waveform of the LFM autocorrelation function, the first lobe value of the dual autocorrelation function is greatly reduced. Similar to the window function processing, the dual autocorrelation function also increases the width of the main lobe and reduces the axial resolution to some extend. While the pulse width of the LFM signal is fixed at 10 μs and the bandwidth varies from 1 to 20 MHz, the first lobe values of the LFM autocorrelation envelope waveform and the dual autocorrelation envelope waveform are shown in Fig. 4c. With the increase of spectrum bandwidth, the first lobe value of the envelope signal of the LFM autocorrelation function rises slightly. The first lobe value of the envelope signal of the dual autocorrelation function decreases first and then increases as the bandwidth of the LFM signal increases. While the bandwidth of the LFM signal increases up to 5 MHz, the first lobe value reaches the minimum of − 30.19 dB. The reason is that the transport channel response is the Gaussian function. As the spectual bandwidth is too large, the final signal is seriously distorted, resulting in the increase of the first lobe value.

Considering the influence of the number of the phased array elements on the detecting signal, the transport channel response between each element and the reflector is assumed to be equal to $h\left( t \right)$, and then from Eq. (21) the final focusing signal is obtained as

25 $$y_{sum}^{TR} \left( t \right) = N \cdot s\left( {t - T_{r} } \right) \otimes s\left( t \right) \otimes h_{i} \left( {t - T_{r} } \right) \otimes h_{i} (t),$$

where $N$ represents the number of the phased array elements. The peak value gain of the phased array compared to the single element is

26 $$G_{peak\_value} = 20 \cdot \log_{10} \left[ {\frac{{N \cdot s\left( t \right) \otimes s\left( {t - T_{t} } \right) \otimes h\left( t \right) \otimes h\left( {t - T_{t} } \right)}}{{s\left( t \right) \otimes s\left( {t - T_{t} } \right) \otimes h\left( t \right) \otimes h\left( {t - T_{t} } \right)}}} \right] = 20\log {}_{10}\left( N \right),$$

which is measured in decibels. The relationship between the number of elements and the peak value gain of the phased array is shown in Fig. 4d. The peak value gain of the phased array is simulated in the FEM, and the results are close to the theoretical value and slightly lower than the theoretical value. The FEM model in Fig. 4d adopts the spatial dimension of the two-dimensional plane. The theoretical model in Eq. (26), which ignores the difference of each array’s transport channel, is the optimal gain theoretically. The transport channel of the middle transmitted transducer is sellected as the reference element, and then the distance between other transmitted transducers and the received transducer is longer. So the peak value gain of the actual transducer arrays cannot reach the prediction in Eq. (26), which is in line with our expectation.

### Adaptive filtering deconvolution method

While the LFM electrical signal is input to one side of a piezoelectric transducer, which is usually used to stimulate the mechanical vibration of the transducer, then the other side of the piezoelectric transducer generates acoustic waves into the measured object for detection. However, because of the bandwidth limitation of the transducer, the output signal of the transducer received is always deformed into a spindle-shaped waveform. In this case, the compressed effect is seriously reduced. In general, the impulse response function of the transducer is approximate to the Gaussian signal in Eq. (23). And time domain expression of the above Gaussian signal can be written as^36^

27 $$s_{tr} \left( t \right) = \frac{{B_{tr} }}{{\sqrt {\upalpha } }}\sqrt {\uppi } e^{{ - \frac{{B_{tr}^{2} }}{{\upalpha }}{\uppi }^{2} t^{2} + j2{\uppi }f_{tr} t}} .$$

In particular, the input LFM signal has the pulse width of 10 μs, the bandwidth of 4 MHz, and the central frequency of 5 MHz and the transducer has the bandwidth of 3 MHz and the central frequency of 5 MHz. The waveform of LFM signal passing through the transducer is expressed as

28 $$s_{out} = s\left( t \right) \otimes s_{tr} \left( t \right).$$

**Figure 5:**
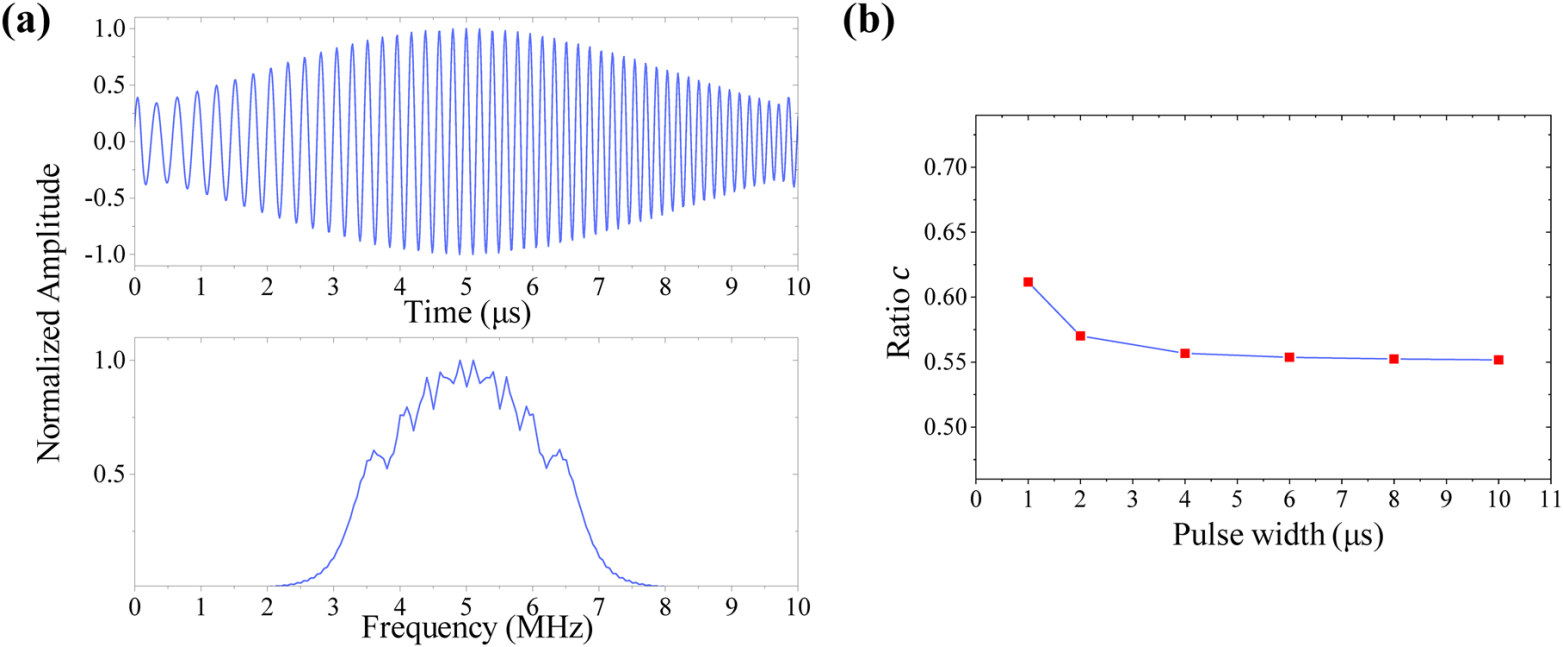
The LFM signal and its corresponding spindle signal. (**a**) The time domain waveform of the spindle signal and its amplitude-frequency characteristic curve. (**b**) The relationship between the ratio *c* of the peak values and pulse width *T*.

The time domain of the signal $s_{out} \left( t \right)$ is shown in Fig. 5a, which deforms into a spindle-sharped signal. The corresponding amplitude-frequency characteristic curve is also provided in Fig. 5a. Defining the expression of ratio $c$ as

29 $$c = \frac{{A_{s} }}{{A_{l} }},$$

where $A_{s}$ and $A_{l}$ represent the peak value of spindle-sharped waveform and LFM waveform after pulse compression respectively. The ratio $c$ is much lower than 1, and it small decreases as the pulse width increases, as shown in Fig. 5b. The peak value of the spindle-sharp signal after pulse compression is obviously smaller than the peak value of the initial LFM signal. Compared with the LFM signal, the input energy and the bandwidth of the spindle-shaped signal are reduced.

In this study, the adaptive filtering method is used to realize the controllable and adjustable detecting signal in ultrasonic detecting. First, according to the actual detecting demand, the waveform of the expected detecting signal is designed in advance. Then, according to the response function of the selected detecting system, the exciting signal corresponding to the detecting signal designed in advance is solved by means of adaptive filtering deconvolution method. Finally, the exciting signal obtained is input to the detecting system by the arbitrary signal generator to get the desired detecting signal.

The detecting system consists of the transmitted transducer, the acoustic medium and the received transducer here. The input exciting signal, the impulse response of the detecting system, and the reveived detecting signal are expressed as $x_{i} \left( n \right)$, $h_{i} \left( n \right)$ and $y_{i} \left( n \right)$, respectively. The subscript $i$ represents the $i$th element of the phased array. The reveived detecting signal is the convolution of the input exciting signal and the impulse response of the detecting system. The time domain model is expressed by sampling discrete sequence as

30 $$y_{i} \left( n \right) = x_{i} \left( n \right) \otimes h_{i} \left( n \right).$$

If the expected detecting signal $y_{i\_\exp ected} \left( n \right)$ of the $i$ th element is determined in advance, then the exciting signal and the impulse response of the detecting system need to be known to obtain the detecting signal $y_{i\_\exp ected} \left( n \right)$.

Inputting a sharp pulse exciting signal $x_{i0} \left( n \right)$ to the certain detecting system and the received signal at the reflector is $y_{i0} \left( n \right)$. The received signal $y_{i0} \left( n \right)$ is re-emitted after time reversal as

31 $$y_{i0\_TR} (n) = y_{i0} ( - n) = x_{i0} ( - n) \otimes h_{i} \left( { - n} \right).$$

**Figure 6:**
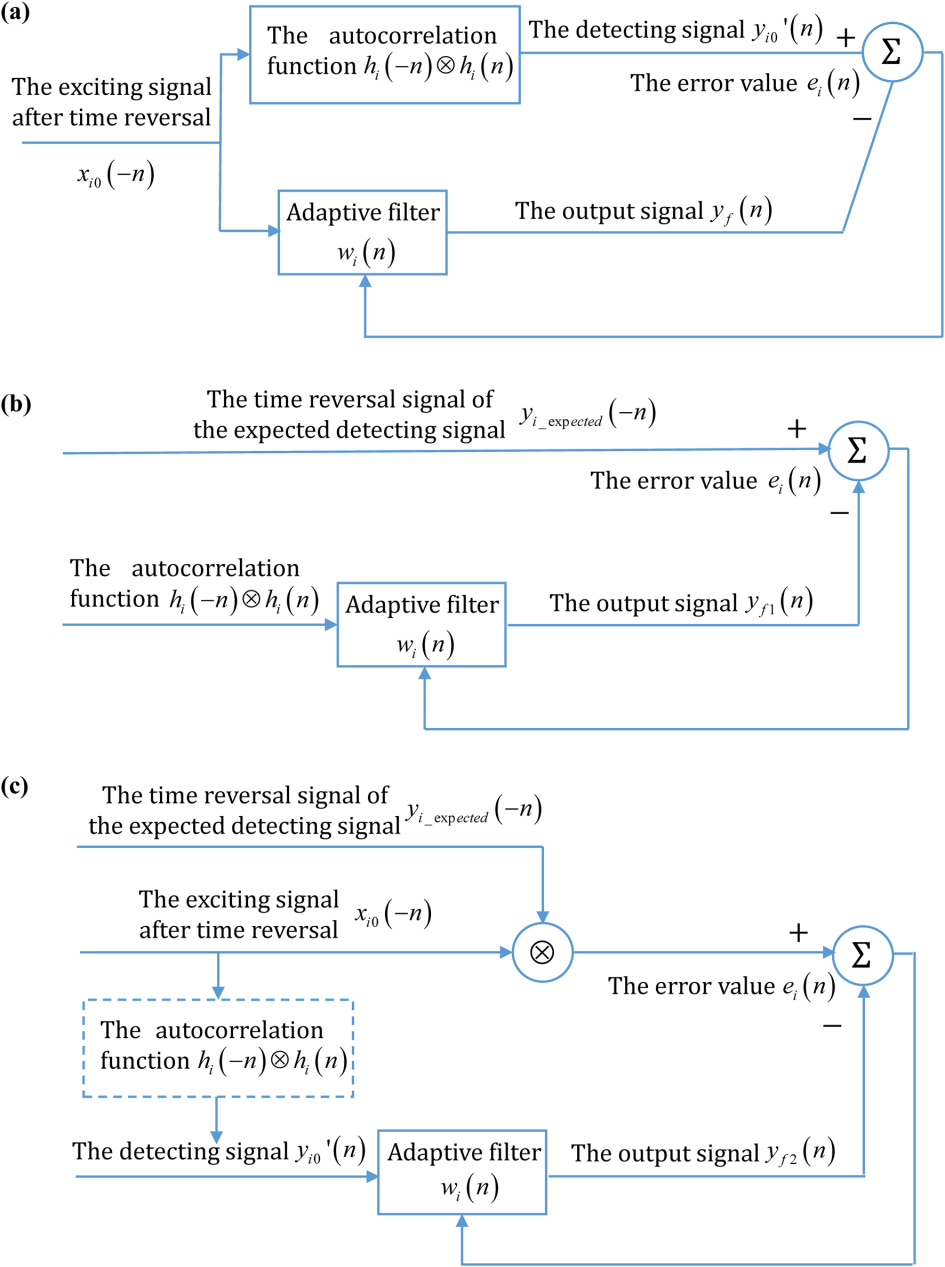
The adaptive filtering deconvolution operation. (**a**) The flow diagram to calculate the autocorrelation function of the detecting system’s impulse response. (**b**) The flow diagram to calculate the exciting signal corresponding to the expected detecting signal. (**c**) The process diagram after the combination.

The signal received secondly at the reflector is

32 $$y_{i0} ^{\prime}(n) = x_{i0} ( - n) \otimes h_{i} \left( { - n} \right) \otimes h_{i} \left( n \right),$$

where the detecting system response function $h_{i}\left( n \right)$ and its time reversal signal $h_{i}\left( -n \right)$ are a pair of time reversal solutions satisfying the same wave equation and the convolution result of them has the autocorrelation property. Collecting this pair of time series data $x_{i0} \left( -n \right)$ and $y_{i0} ^{\prime}\left( n \right)$, and then the autocorrelation function of the detecting system’s impulse response is obtained by means of adaptive deconvolution as

33 $$R_{hh} (n) = h_{i} \left( { - n} \right) \otimes h_{i} \left( n \right) = y_{i0} ^{\prime}(n) \otimes^{ - 1} x_{i0} ( - n),$$

where the $\otimes^{ - 1}$ represents the deconvolution operation. $y_{i0} ^{\prime}\left( n \right)$ is regarded as the reference output of the adaptive filter and $x_{i0} \left( { - n} \right)$ is regarded as the input signal of the adaptive filter. The actual output $y_{f} (n)$ of the adaptive filter is the convolution of $x_{i0} \left( { - n} \right)$ and the weight sequence $\omega_{i} \left( n \right)$ of the adaptive filter. According to the error value $e_{i} \left( n \right)$ between the actual output $y_{f} (n)$ and the reference output $y_{i0} ^{\prime}\left( n \right)$, the adaptive filter regulates the weight sequence by a certain algorithm to control the approximation of $y_{f} (n)$ to $y_{i0} ^{\prime}\left( n \right)$, so as to obtain the optimal weight sequence of the adaptive filter $w_{i}^{*} \left( n \right) = h_{i} \left( { - n} \right) \otimes h_{i} \left( n \right)$. The operation process is shown in Fig. 6a.

The expected detecting signal $y_{i\_\exp ected} \left( n \right)$ of the $i$ th element is determined in advance according to the detecting requirements and then the corresponding exciting signal is expressed as

34 $$x_{i\_\exp ected} \left( n \right) = y_{i\_\exp ected} \left( { - n} \right) \otimes^{ - 1} \left[ {h_{i} \left( { - n} \right) \otimes h_{i} \left( n \right)} \right].$$

The adaptive filtering is used for deconvolution again to control the output signal $y_{{f{1}}} \left( n \right)$ of the adaptive filter to approximate $y_{i\_\exp ected} \left( { - n} \right)$, which obtains the optimal weight sequence of the adaptive filter $w_{i}^{*} \left( n \right) = x_{i\_\exp ected} \left( n \right)$. The operation process is shown in Fig. 6b.

Simultaneous the Eqs. (33) and (34) gives

35 $$x_{i\_\exp ected} \left( n \right) = \left[ {x_{i0} \left( { - n} \right) \otimes y_{i\_\exp ected} \left( { - n} \right)} \right] \otimes^{ - 1} y_{i0} ^{\prime}\left( n \right).$$

While the error $e_{i} \left( n \right)$ is small enough, the optimal weight sequence of the adaptive filter is $w_{i}^{*} \left( n \right) = x_{i\_expected} \left( n \right)$. After merging the previous two steps, only one time operation of the adaptive filter is needed to obtain the exciting waveform corresponding to the designed detecting signal. The operation process is shown in Fig. 6c. Firstly, the adaptive filter needs to select a certain algorithm, and then the corresponding exciting signal waveform can be calculated according to the pair of time series data of the exciting signal and the detecting signal collected in practice. In this way, the detecting signal at the reflector of a single element can be designed in advance. The focusing result with the standard LFM signal can be obtained by applying the adaptive filtering method for each element in turn, and then the superposition signal of the Eq. (22) becomes

36 $$y_{sum\_A}^{TR} \left( t \right) = \left\{ {\begin{array}{*{20}c} {N \cdot \sqrt {B \cdot T} \cdot rect\left( {\frac{{t - T_{r} }}{2T}} \right)Sa\left( {{\uppi }B\left( {t - T_{r} } \right)} \right)\left( {\left| {t - T_{r} } \right| \le T} \right)} \\ {0\left( {\left| {t - T_{r} } \right| > T} \right)} \\ \end{array} } \right..$$

The superposition signal obtained by the adaptive filtering method is the autocorrelation function of the standard LFM signal, which is able to further improve the controllability and SNR of the focusing signal. The adaptive filtering deconvolution method, which is also applicable well to the transport channel response of other waveforms, is a universal method. When the ultrasonic phased array has the self-transmitted and self-received detecting mode, the adaptive filtering deconvolution method is also capable of regulating the waveform of the detecting signal. An array element of the phased array is excited firstly, and each array element receives its corresponding echo signal of the reflector. Then the received signal is excited by each array element in turn after time reversal and later reveives its corresponding second echo signal of the reflector again. Collecting this pair of time series data of the first excited signal and the second received signal. By imitating the solving menthod in Eq. (35), the excited signal waveform $x_{i\_\exp ected} \left( n \right)$ corresponding to the expected detecting signal $y_{i\_\exp ected} \left( n \right)$ can be obtained. The adaptive filtering deconvolution method has good generality in controling the waveform of the detecting singal.

## Conclusions

In summary, we demonstrate autocorrelation property of the TRM from the time-reversal symmetry of the wave equation and establish the theoretical model of TRM method based on the standard LFM signal. Applying the LFM signal as the exciting signal to the TRM, the received signal is the dual autocorrelation function of the exciting signal and the transport channel response. With the increase of the pulse width and bandwidth of the LFM signal, the peak value and the axial resolution of the compressed pulse signal can be improved significantly. The peak value of the final superimposed signal in TRM depends on the pulse width of the LFM exciting signal and the number of the array elements actually. The theoretical model predicts that the peak value optimal gain of the TRM’s final focusing signal is 26.02 dB as the number of the array elements increases to 20. According to the impulse response of the detecting system, we adopt the adaptive filtering deconvolution method to regulate the input signal, which can ensure that the detecting signal is the standard LFM signal designed in advance and further enhance the matched filtering effects. It is expected that the LFM signal would be applied to the TRM technology, and then the adaptive filtering deconvolution method is used to regulate the input signal, so as to promote the further applications of the TRM technology.

## Supplementary Information

Supplementary Information.
