# Supplementary material for: An improved time reversal mirror based on standard linear frequency modulation waveform
Source: Sci Rep. 2021 Jan 8;11:194. doi: 10.1038/s41598-020-79884-w (PMC7794588; doi:10.1038/s41598-020-79884-w)
Supplement: Supplementary file 1 — Supplementary Information. [file 41598_2020_79884_MOESM1_ESM.docx]

Supporting Information

**Time Reversal Acoustic Mirror Technology Based on the Standard Linear Frequency Modulation Signal**

Yongkang Wang^1,5^, Han Zhang^2,3,4*^, Huiling Li^1,5^, Jianfeng Zheng^6^ & Liang Guo^1^

^1^State Key Laboratory of Nonlinear Mechanics, Institute of Mechanics, Chinese Academy of Sciences, Beijing 100190, China.

^2^Key Laboratory of Noise and Vibration, Institute of Acoustics, Chinese Academy of Sciences, Beijing 100190, China.

^3^State Key Laboratory of Acoustics, Institute of Acoustics, Chinese Academy of Sciences, Beijing 100190, China.

^4^University of Chinese Academy of Sciences, Beijing 100049, China.

^5^School of Engineering Science, University of Chinese Academy of Sciences, Beijing 100049, China.

^6^School of Mechanical Engineering, Changzhou University, Changzhou 213164, China.

*Corresponding author. E-mail: zhanghan@mail.ioa.ac.cn

**Section S1. Phase conjugation and time reversal**

The Fourier transform of the time domain solution $p\left(r,t\right)$ gives the frequency domain solution $p\left(r,\omega\right)$ of the acoustic pressure field

$p\left(r,\omega\right)=\int_{-\infty}^{+\infty} p\left(r,t\right)e^{-j\omega t}dt$ (S1)

The inverse Fourier transform is

(S2)

The conjugate result of acoustic pressure field $p\left(r,t\right)$ is expressed as $p^{*}\left(r,t\right)$, and the Fourier transform of which is

(S3)

If $p\left(r,t\right)$ only contains the real part, then

$p\left(r,t\right)=p^{*}\left(r,t\right)$ (S4)

Substitution of equation (S4) into equation (S3) gives

, (S5)

which is simply transformed into

$p\left(r,-\omega\right)=p^{*}\left(r,\omega\right)$ (S6)

For the time-reversal solution $p\left(r,-t\right)$, the frequency domain solution of the acoustic pressure field is

(S7)

Substitution of equation (S6) into equation (S7) gives

(S8)

**Section S2. Autocorrelation processing**

The autocorrelation function of signal $s\left(t\right)$ is

$R_{ss}\left(t\right)=s\left(t\right)\otimes s^{*}(-t)=\int_{-\infty}^{+\infty} s\left(\tau\right)s^{*}\left(\tau-t\right)d\tau$, (S9)

and $s\left(t\right)$ only contains the real part gives

$R_{ss}\left(t\right)=s\left(t\right)\otimes s(-t)=\int_{-\infty}^{+\infty} s\left(\tau\right)s\left(\tau-t\right)d\tau$ (S10)

While the signal $s\left(t\right)$ mixed noise signal $n\left(t\right)$, the autocorrelation processing gives

$R_{f}\left(t\right)=\int_{-\infty}^{+\infty} \left[s\left(\tau\right)+n(t)\right]\left[s\left(\tau-t\right)+n(\tau-t)\right]d\tau=R_{ss}\left(t\right)+R_{sn}\left(t\right)+R_{ns}\left(t\right)+R_{nn}\left(t\right)$ (S11)

There is almost no correlation between noise signal $n\left(t\right)$ and deterministic signal $s\left(t\right)$, so $R_{sn}\left(t\right)\approx0$ and $R_{sn}\left(t\right)\approx0$ are derivable. In general, the intensity of autocorrelation function $R_{nn}\left(t\right)$ of noise signal is very small and decay fast, so we can get $R_{f}\left(t\right)\approx R_{ss}\left(t\right)$. Since the noise is greatly suppressed, the autocorrelation function of mixed signal is close to the autocorrelation function that deterministic signal.

**Section S3. Matched filtering**

The matched filter is the linear filter that maximizes the SNR of the received signal. The input signal of the filter is expressed as

$r\left(t\right)=s\left(t\right)+n\left(t\right)$, (S12)

where $s\left(t\right)$ and $n\left(t\right)$ represent the symbol signal and noise signal. The bilateral power spectral density of the noise signal is $n\left(t\right)$. The output signal after filter is

$r_{0}\left(t\right)=s_{0}\left(t\right)+n_{0}\left(t\right)$ (S13)

The average power of the output noise signal is defined as

$N_{0}=\frac{n_{0}}{4\pi}\int_{-\infty}^{+\infty} \left|G(\omega)\right|^{2}d\omega$, (S14)

where $G(\omega)$ is the Fourier transform of the impulse response function of the matched filter. At the sampling time $t_{0}$, the ratio of the instantaneous power of the output signal to the average power of the noise is

$SNR=\frac{\left|s_{0}\left(t_{0}\right)\right|}{N_{0}}=\frac{\left|\frac{1}{2\pi}\int_{-\infty}^{+\infty} G\left(\omega\right)S\left(\omega\right)e^{j\omega t_{0}}d\omega\right|^{2}}{\frac{n_{0}}{4\pi}\int_{-\infty}^{+\infty} \left|G\left(\omega\right)\right|^{2}d\omega}$, (S15)

Schwartz inequality gives

$\frac{\left|\frac{1}{2\pi}\int_{-\infty}^{+\infty} G\left(\omega\right)S\left(\omega\right)e^{j\omega t_{0}}d\omega\right|^{2}}{\frac{n_{0}}{4\pi}\int_{-\infty}^{+\infty} \left|G\left(\omega\right)\right|^{2}d\omega}\leq\frac{2\times\left|\frac{1}{2\pi}\int_{-\infty}^{+\infty} \left|S\left(\omega\right)\right|^{2}d\omega\right|}{n_{0}}=\frac{2\times\int_{-\infty}^{+\infty} s\left(t_{0}\right)^{2}dt}{n_{0}}=\frac{2E}{n_{0}}$ (S16)

The inequation (S16) is equal while

$G\left(\omega\right)=kS^{*}\left(\omega\right)e^{-j\omega t_{0}}$ (S17)

The impulse response function of the matched filter is

$g\left(t\right)=\frac{1}{2\pi}\int_{-\infty}^{+\infty} G\left(\omega\right)e^{j\omega t}d\omega=\frac{1}{2\pi}\int_{-\infty}^{+\infty} kS^{*}\left(\omega\right)e^{j\omega\left(t-t_{0}\right)}d\omega=ks^{*}\left(t_{0}-t\right)$ (S18)

In general, $k=1$, $t_{0}=0$ and $s^{*}\left(t\right)=s\left(t\right)$ gives

$g\left(t\right)=s\left(-t\right)$ (S19)

The output signal of the signal $s\left(t\right)$ after passing through the matched filter is

$s_{0}\left(t\right)=s\left(t\right)\otimes s\left(-t\right)$, (S20)

which is equal to the autocorrelation function of the signal $s\left(t\right)$.

**Section S4. The time bandwidth product *D* of the traditional signals**

The Fourier transform of $s\left(t\right)$ is expressed as $S\left(j\omega\right)$, then the Fourier transform of $s\left(at\right)$ is

(S21)

$x=at$ gives

$dt=\frac{1}{a}dx$ (S22)

While $a>0$,

(S23)

While $a<0$,

(S24)

So the Fourier transform of $s\left(at\right)$ can expressed as

(S25)

The equation (S25) indicates that the signal $s\left(t\right)$ is compressed to $1/a$ on the timeline while its corresponding spectrum expands $a$ times on the $\omega$ axis. It can be concluded that the time bandwidth product of traditional single carrier frequency signals is a constant.
